# Does a Different Household Registration Affect Migrants’ Access to Basic Public Health Services in China?

**DOI:** 10.3390/ijerph16234615

**Published:** 2019-11-20

**Authors:** Xia Meng

**Affiliations:** School of Economics and Management, China University of Geosciences (Wuhan), Wuhan 430074, China; mengxia@cug.edu.cn; Tel.: +86-139-7125-1305

**Keywords:** household registration, agricultural Hukou and non-agricultural Hukou, migrant populations, basic public health services

## Abstract

On the basis of the China Migrants Dynamic Survey Data of 2015, the author provides an analysis of how a different household registration impacts migrants’ access to preventive care provided by public health services, such as health records and medical knowledge, in areas of immigration. This study shows that eliminating the distinction between agricultural and non-agricultural permanent residence registration could raise the rate of establishing health files, but it has no significant effect on migrants’ health knowledge. In fact, encouraging those with non-agricultural registration to move to different counties that belong to the same city or to different cities that belong to the same province can notably eliminate the impact of a different household registration status. Improving the income level of low-income migrants can have the same impact. Recommendations to enable migrants to obtain basic public health services include abolishing the separation of agricultural and non-agricultural household registration, increasing the permanent settlement rate of resident migrants, promoting basic medical security systems across the whole country, strengthening career training, and enhancing the education level of migrants.

## 1. Introduction

Since the reform and opening-up, the scale of China’s floating population has been increasing, making an important contribution to promoting China’s urban construction and economic and social development. By the end of 2018, the number of Chinese households that lived out of their place of permanent registration reached 286 million, including a floating population of 241 million. Under the current fiscal system and the public service supply mechanism in China, local governments generally allocate public service resources, such as basic public health, in their administrative jurisdictions according to the registered population of the jurisdiction, which yields obvious spatial and regional heterogeneity and entails “competition” and “exclusion” for nonlocal households from outside the jurisdiction. The Chinese household registration system has long been an important institutional basis for the allocation of urban and rural public services. The divided household registration system in urban and rural areas makes it difficult for migrants entering the city to obtain the same public services and social benefits as the registered population. Different household registrations correspond to different levels of social welfare, resulting in segregation and discrimination by household registration status [1,2,3]. This is reflected in the separation of household registration status between urban and rural areas, between local and nonlocal households within a city, between migrants with agricultural and non-agricultural hukou, and, finally, between urban floating populations with agricultural and non-agricultural household registration. The different access to public services caused by a difference in household registration has been widely confirmed, while the resulting public service discrimination has not been thoroughly studied.

Reform of the household registration system is an important institutional change in China that would improve the allocation of urban and rural resources and achieve the equalization of basic public services. Eliminating the discrimination associated with household registration would accelerate the flow of talents and human resources, embody the values of social fairness and justice, and stimulate a new round of growth in China’s economy [4]. In 2014, the State Council issued “The Opinions of the State Council on Further Promoting the Reform of the Household Registration System”, which proposed to abolish the distinction between “agricultural” and “non-agricultural” household registration, further adjust the household registration policy, and unify the urban and rural household registration systems to expand the target requirements of public service coverage. By 2016, all provinces (cities) in China had introduced implementation plans for the reform of the household registration system and followed the target requirements of this reform. Since then, the State Council has successively issued “Several Opinions on Deepening the Construction of New Urbanization” and “Promoting 100 Million Non-Resident Population in Urban Set-up Program” to further accelerate the implementation of the policy reform of the household registration system and promote the equalization of basic public services. Can removing the distinctions of urban migrants and non-agricultural household registration help to improve the level of basic public health services available to nonlocal households? Does the division by household registration affect the urban migrant population’s access to basic public health services? How do local governments react to a new situation of basic public health resource allocation? On the basis of the data of China’s Floating Population Health Plan Dynamics Monitoring Survey, in 2015, this study quantitatively evaluates whether there are differences in access to basic public health services between urban households with agricultural and non-agricultural household registration, and conducts an in-depth study of the household registration factors and other factors in obtaining basic public health for migrants. This study of the fairness of service provision provides a basis for decision making in promoting the reform of China’s household registration system and the full coverage of public health services.

Foreign scholars have conducted in-depth research on the public nature of access to local health services based on differences in immigration and resident status [5,6,7]. Immigration status and family characteristics are generally considered important factors. For example, in Denmark, Nieisen et al. [8] studied the differences in the use of medical resources between immigrants and their offspring and people of Danish ethnicity. Studies have shown that the use of free medical services does not indicate systematic inequity, but for dentists, who require fees, the use of services by immigrants and their descendants shows significant inequity as compared to that of local Danish residents. García-Pérez [9] follows the Behavioral Model of Health Service Use [5], which analyzes the differences in access to health care resources between nonimmigrant children and immigrant children of different nationalities, considering the immigration status of the parents and children. Immigration status has a significant impact on the access of immigrant children to health care, especially for noncitizen children of immigrant families. In China, because of the household registration system, urban and rural residents have differential access to social welfare [10,11,12,13]. Urban residents and their children enjoy education, medical care, social security, and other aspects of social welfare which rural residents and their children do not enjoy [14]. For example, Yao et al. [15] believed that China’s urban-rural dual structure implies not only a different household registration (non-agricultural and agricultural) but also different resource allocation methods. There are inherent differences in the fairness and accessibility associated with the dual economic structure of the non-agricultural and agricultural household registration system. On the basis of the comparative analysis of Shanghai’s registered population and floating population, Tao and Shen [16] believed that the registered population has better accessibility than the floating population. The difference between the two groups is most obvious in the central city area and the suburbs. Ma et al. [17] analyzed migrants’ unequal access to medical insurance in the context of urban and rural medical insurance. Studies have shown that, under both principles of ex ante compensation and ex post compensation, the agricultural households that participate in the new rural cooperatives face significantly unequal opportunities due to their household registration. In addition to immigration or nonimmigrant status and household registration factors, nonresidential factors, such as income status, years of education, and whether insurance is purchased, have been investigated by various scholars.

The existing literature mainly focuses on the unfairness between migrants and nonmigrants, between urban and rural residents, and between urban local household registration and other resident registration in benefiting basic health service. Few studies have considered migrants with agricultural permanent residence identity and those with non-agricultural permanent residence identity, and therefore there is no evidence whether permanent residence identity has an impact on the benefits to migrants of the government’s basic health service. Household registration status is an important proxy variable of social resources [18]. Local governments have largely referred to macro household registration policies and household registration information in the allocation of public resources in the region and have canceled “agricultural” and “non-agricultural” household registration. It remains unclear whether the distinction between registration identities has a substantial impact on the city’s basic public health service provision to the floating population. The main contributions of this study are as follows: First, it presents an in-depth analysis of the differences in household registration status among urban migrants and investigates whether these differences affect the fair access to urban basic public health services. Secondly, it examines the role of non-household factors to further promote the reform of the household registration system and equalize public services, and provides new practical evidence and policy recommendations.

The remainder of the paper is structured as follows: the second section describes the data, variables and models, the third section analyzes the empirical results, the fourth section presents the robustness test, and the last section concludes the paper.

## 2. Materials and Methods

### 2.1. Data Sources

This study used data from the China Migrants Dynamic Survey (CMDS), which was published by the National Health Commission of the People’s Republic of China, in 2015. The survey took the annual report data concerning the migrant population of 31 provinces (autonomous regions and municipalities) and Xinjiang Production and Construction Corps, in 2014, as the basic sampling frame. The sampling methods included stratified random sampling, multistage sampling, and probability proportional to size sampling (PPS). The respondents were the inflow population that had lived in the destination for more than one month, were aged 15 and older, and were not registered in the district (county or city). The survey information covered the basic characteristics of the migrants and their family members, as well as employment, basic public healthcare, and family planning policy services. This study took the migrant population with “local” current residence and aged 15 to 60 years old as the research object. According to the research needs of this study, samples with missing variables were eliminated to obtain an effective research sample size of 59,443, covering 31 provinces (autonomous regions and municipalities) and Xinjiang Production and Construction Corps.

### 2.2. Main Variables

#### 2.2.1. Dependent Variable

This study focused on the impact of differences in the household registration status of the migrant population on the equity of access to urban basic public healthcare services. Healthcare services were divided into preventive care and therapeutic care services. The former included routine physical examination, vaccination, health records, and health education, and the latter included outpatient visits, emergency rates, and inpatient medical services [19,20]. On the basis of the available survey data, the basic public healthcare services studied refer to preventive care services. To fully reflect the basic public healthcare services available to the migrant population in cities, this study conducted a regression analysis of two basic public health service variables: (1) the establishment of residents’ health records in their living communities [21], where the value 1 indicated the establishment of health records, and 0 otherwise; and (2) access to health-related knowledge in inflow areas in the past year, which mainly included the prevention and control of occupational diseases, chronic diseases, tuberculosis, STD and AIDS, mental disorders, contraception and eugenic birth, and prevention and control of infectious diseases, where the value 1 indicated access to knowledge of at least one of these items, and 0 otherwise.

#### 2.2.2. Independent Variables and Control Variables

The core independent variable of this study was the dummy variable of household registration. The migrant population with agricultural household registration received the value 0, and that with non-agricultural household registration received the value 1. This study also controled other important variables affecting migrants’ access to basic public healthcare services to alleviate missing variables which included: (1) individual characteristics of the migrant population, such as gender, age, marriage, education level, employment status, type of mobility, time of residence in the inflow area, social medical insurance status, and residence willingness; (2) family characteristics of the migrant population, such as the total monthly household income in the past year (after tax); and (3) regional characteristics according to the national regional development strategy, where 31 provinces (cities) are divided into the eastern, central, western, and northeast regions.

**Figure 1:**
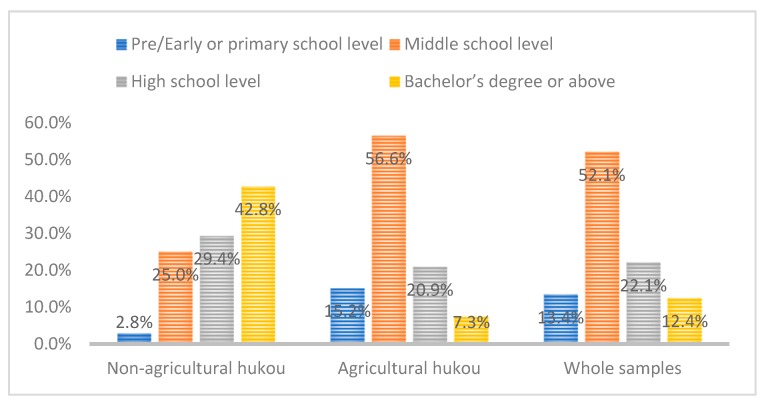
Education levels of various samples.

**Figure 2:**
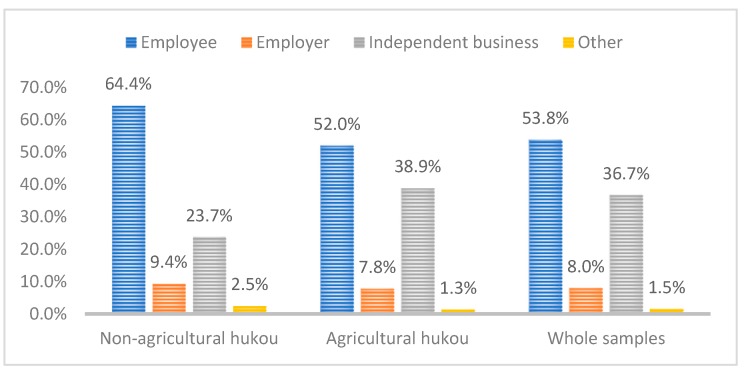
Employment status of various samples.

According to Table 1, migrants with agricultural household registration and non-agricultural household registration accounted for 85.7% and 14.3% of the total sample, respectively. The differences between the two groups in terms of individual characteristic variables, such as age, education level, and employment status, are significant at the 1% level. In the research sample, people with bachelor’s degrees comprise 42.8% of the migrants with non-agricultural registered permanent residence identity, and 64.4% of them are employed. Among the migrants with agricultural permanent residence identity, those with middle school education represent 56.6%, and 52% are employed. Moreover, there are significant differences in the migration flow patterns. The gap in the mean values of different income levels remains large. These research outcomes suggest that permanent residence identity has strong links with these variables (Figure 1 and Figure 2).

**Figure 3:**
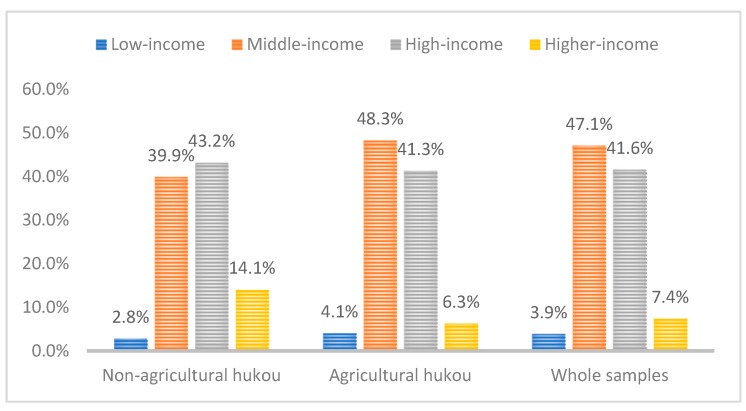
Proportion of different income levels of various samples.

In addition, there are significant gaps between different flow patterns and income levels, which means that household registration factors may have strong links with these variables. The majority of migrants with non-agricultural permanent residence identity are in a higher income class, accounting for 43.2% of the total. In contrast, the majority of migrants with agricultural permanent residence identity are in the middle income class, accounting for 48.3% of the total (see Figure 3). In China, the household registration system is deeply rooted and is closely related to welfare system arrangements and public goods supply. Household registration and non-household registration factors have mutual impacts on each other, so their interaction must be analyzed.

### 2.3. Model

To examine the impact of differences in household registration on the basic public healthcare of the migrant population, this study used logistic regression to estimate the two types of basic public healthcare service variables established above.

$$P\left(Y=1\right)=F\left(\alpha_{0}+\alpha_{1}hou_{i}+\alpha_{2}X_{i}+\alpha_{3}family+\alpha_{4}region+u_{i}\right)$$

Assume that the conditional probability of an event occurring is:

$$P\left(Y=1\right)=p_{i}$$

The logistic regression model is:

$$Ln\left(\frac{p_{i}}{1-p_{i}}\right)=\alpha_{0}+\alpha_{1}hou_{i}+\alpha_{2}X_{i}+\alpha_{3}family+\alpha_{4}region+u_{i}$$

where F(·) is the logistic cumulative distribution function, i denotes a migrant, p is the probability of an event occurring, hou indicates the household registration characteristics of the migrant, and X_it_ represents a vector of migrant I’s basic demographic characteristics, family and region, accounting for family characteristics and region characteristics.

## 3. Results

### 3.1. Factor Analysis

Table 2 presents the results of the regression of the interpreted variables, y1 and y2, which yield two findings.

On the one hand, when controlling other variables, household registration has a significant impact on whether a migrant has established a health record, but the impact on whether the migrant has access to health knowledge is not statistically significant. This indicates that household registration has a significant effect on migrants’ access to preventive health services as follows: (1) Migrant households with non-agricultural registration have a higher probability of establishing health records than agricultural households, and the difference is significant at the level of 5%. Furthermore, the odds ratio for migrants with non-agricultural household registration is 1.1 times that of migrants with agricultural registration. This shows that eliminating the distinction between agricultural and non-agricultural household registration can significantly improve the construction of health records for the floating population. (2) There is no statistical impact on the access to health knowledge concerning the prevention and treatment of various diseases among migrants based on their household registration. This shows that migrants’ access to health knowledge, such as occupational and chronic disease prevention, is not affected by the nature of their household registration. A possible reason is that the difference between agricultural and non-agricultural household registration is mainly reflected in the availability of medical resources, fairness and the cost burden [15]. With the improvement of China’s basic public service system, the migrant population has increasingly extensive access to health knowledge. According to the questionnaire, 84.3% and 80.3% of the sampled migrants obtained health knowledge through radio and television programs and billboards, respectively, and 58.2% obtained health knowledge through mobile phone text messages and WeChat, books and CD-ROMs. The vast majority of the migrant population, 78.6%, obtained health knowledge through three or more channels, yielding significant spillover effects. The increase in the accessibility and equity of health knowledge for the migrant population, as well as the reduction of access costs, eliminate the impact of different household registration status. (3) By category, the basic public health services with significant spillover and high accessibility do not present differences according to household registration status, while those provided through community hospitals or government financial subsidies do.

On the other hand, individual characteristics, family characteristics, and non-household factors also affect the access of the floating population to basic public health services. Those with nonlocal and agricultural household registration generally find it more difficult to enjoy complete urban public benefits than those with local and urban household registration, and therefore are defined as having “vulnerable household registration” [22]. Households’ vulnerability to discrimination by virtue of their registration is also affected by individual characteristics. Age, marital status, education level, occupation type, mode of migration, length of residence, and family income, etc., have a significant impact on the access of the floating population to basic public health services as foolows.: (1) The influence of individual characteristics, such as gender, marital status, age, education level, and length of residence, on the two interpreted variables showed consistency. For example, men in the migrant population are less likely than women to establish health files and acquire health knowledge. The incidence of establishing health files and acquiring health knowledge among the migrants with high school education was 1.23 times and 1.27 times higher than the incidence among those with primary school education, and the incidence among migrants having lived three to six years in their current location was 1.12 times and 1.11 times the incidence among migrants with less than three years of residence. (2) The effects of individual characteristics, such as flow pattern, employment status, and income level, on the two interpreted variables are inconsistent. For example, a cross-provincial migrant is less likely to establish a health file than a cross-county migrant with urban registration. However, cross-provincial migrants are 1.1 times more likely than cross-county urban migrants to access health knowledge. Migrants with high income are less likely to establish health files than those with low income (OR = 0.884), while migrants with high income have higher odds to acquire health knowledge than those with low income (OR = 1.943). (3) Participation in health insurance has a significant impact on y1 and y2, and health insurance significantly promotes the use of health services [23]. The incidence of health file establishment and health knowledge access among migrants with health insurance was 1.2 times and 1.3 times higher than the incidence among migrants without health insurance, respectively.

### 3.2. Impact Path Analysis

The above conclusions present significant differences in the impact of household registration and individual characteristics on migrants’ access to basic public health services. Will this difference caused by the zoning of household registration be alleviated or offset by changes in migrants’ education, income levels, and mobility patterns? In this section, we introduce the cross-terms of household registration with education level, income level, and mobility into the model for analysis.

As shown in Table 3, concerning the establishment of health records for migrants, only the cross-terms of “interprovincial*Hou” and “middle income*Hou” are significant at the levels of 10% and 5%, respectively; the other items are not statistically significant. Specifically, the household registration odds ratio (OR) of establishing health records for the cross-city migrants within the province is 0.878 times that of the cross-county migrants within the city. This suggests that household registration has a significant impact on establishing health records. A possible reason is that under the current financial and public service supply system in China, local governments generally allocate public service resources, such as medical and health care, in their administrative jurisdiction according to the population of the district, resulting in obvious spatial and regional heterogeneity, and the residents who seek public service resources outside their place of registration face certain competition and exclusivity. The access to basic public services by household registration status presents relatively small differences within the same province, especially among counties in the same city, and the “hukou discrimination” of the floating population in terms of basic public health services is small. The household registration odds ratio(OR) of high-income migrants to establish health records is 1.388 times that of the low-income migrants, which indicates that middle-income non-agricultural households are more likely to establish health records than low-income non-agricultural households. Therefore, promoting the cross-county mobility and raising the monthly income of non-agricultural households can effectively increase the establishment of health files. However, the improvement of the education level of the migrant population will not automatically eliminate the difference in the establishment of health files.

The other interactions do not have significant effects on migrants’ acquisition of health knowledge. First, as compared to residents, the migrants pay more attention to issues such as family income and children’s education than to a personal healthy situation. Second, due to the diverse means and spillovers of health knowledge access, different household registration status “affection to the accessibility and fairness of migrants” health knowledge is limited. The path of influence mainly lies in improving the education level and income status of the floating population.

## 4. Robustness Analysis

The results of the regression of Table 2 show that the impact of household registration and non-household registration factors on the establishment of health files among the floating population is very significant, but there are obvious differences in the impact of individual characteristics. Do different individual characteristics affect the nature of the household registration of the floating population? This makes us wonder whether factors other than household registration, which are control variables, are truly well controlled, and whether the problem of self-selection bias may exist.To address endogeneity and check the robustness of the regression results, a matching method is used to control the self-selection problem of hukou.

In the analysis above, hukou, education level, employment status, and other non-household factors are controlled in the regression. Whereas, in China, individual characteristics such as education level and employment status strongly affect the individual’s household registration choice, therefore, migrants of different household registrations can present differences in education level, employment status, and other variables, which in turn will affect the regression result.

Therefore, this study adopts the Wei [24] matching method to address the potential sample selection bias and endogeneity. We selected all the individual characteristic variables, except household registration, as the matching variables, and the regression results of the first-stage logit model were significant. This initially proves the important effect of individual characteristics on household registration. Next, each household registration sample (hou = 1) was matched with a similar sample with a different registration status (hou = 0). The regression coefficient of these two groups of samples was still significant, and the conclusion was robust (omit regression results).

The one-to-one nearest neighbor matching method was used. In this study, 8506 control groups and 8506 treatment groups of hou 1 were matched for regression. After the regression, the coefficient of hou increased significantly, and it remained significant because other individual characteristics were eliminated from the effect of the variable y1 by the inclusion of hou. To ensure the robustness of the results, we also used another matching method, Mars matching. The average treatment effect on the treated (ATT) was obtained by matching each observation with the four nearest neighbors using the heteroscedastic-robust variance estimators proposed by Abadie and Imbens [25]. Table 4 shows that after matching, the ATT of hou for y1 was significant at the 10% level, indicating that the abovementioned regression results are stable; different household registration status has a significant impact on y1, and self-selection issues have little impact on the results.

## 5. Conclusions

On the basis of the China Migrants Dynamic Survey Data of 2015, the author provides an analysis of how different household registration impacts migrants’ access to basic public health services, such as building health records and accessing medical knowledge, in immigration areas. Data from China’s Floating Population Health Plan Dynamics Monitoring Survey, in 2015, are used to analyze the impact of household registration status and non-household factors on preventive health services. The main findings of this study are as follows: First, different household registration status presents significantly different impacts on migrants’ access to preventive care. Subject to other conditions, having a non-agricultural hukou will significantly enhance the establishment of health records by migrants, but there is no statistically significant impact on their access to health knowledge. Second, nonregistration factors, particularly education level, income status, mobility pattern, and purchase of medical insurance, have a significant impact on migrants’ access to preventive care. Third, the impact of the household registration on the establishment of health records will be alleviated or offset as the proportion of non-agricultural households migrating across the county but within a city and the income level of low-income migrants increase.

At present, the distinction between agricultural and non-agricultural household registration, in China, has been removed and replaced with unified registration. For migrants, the elimination of household registration differences can eliminate its impact on the fair access to some basic public health services. To further improve the access of the floating population to preventive care services, we should focus on the following two aspects: (1) Household registration factors remove the institutional barriers of household registration system to the migrant’s fair access to preventive basic public health services. The household urbanization rate of the floating population should be improved to encourage more migrants to settle in the city. On the basis of the carrying capacity of economic and social development, each city should optimize the settlement policy of migrants to promote more people to integrate into the city, and the reasonable distribution of public resources and public services between cities and regions, which may also reduce the discrimination with regard to basic public health services. (2) Non-household registration factors, first, is to improve a migrant’s ability of accessing basic public health services. As different groups of migrants have different preventable basic public health service needs, the local government should strengthen health education for migrants and try to strengthen their health awareness and enhance health literacy, and build various platforms for basic public health services for migrants and give more innovation to service models. Secondly, is to improve the policy system of floating population’s access to basic public health services. The government should promote, in all relative areas of society such as building a basic medical security system for all people, increasing the medical insurance participation rate and mutual recognition between different places.

## Figures and Tables

**Table 1:** Sample definitions and descriptive statistics.

Variable	Variable Definitions	Total Sample		Agricultural Household Registration		Non-Agricultural Household Registration		Difference between the Two Groups of Samples
		Mean	SD	Mean	SD	Mean	SD	
y1	Health record established (= 1; else = 0)	0.36	0.481	0.36	0.48	0.38	0.486	−0.024 ***
y2	Health knowledge learned in the inflow area (= 1; else = 0)	0.95	0.214	0.95	0.215	0.96	0.207	−0.004 *
Gender	Male (= 1,female = 0)	0.58	0.494	0.58	0.494	0.57	0.495	0.008 ***
Marital status	Marital status (married = 1; other = 0)	0.80	0.4	0.81	0.394	0.75	0.431	0.053 ***
Age	Age (15–60)	34.98	9.279	34.92	9.343	35.32	8.875	−0.399 ***
Education level	Pre/early or primary school level (= 1; other = 0)	0.13	0.341	0.15	0.359	0.03	0.165	0.124 ***
	Middle school level (= 1; else = 0)	0.52	0.50	0.57	0.496	0.25	0.433	0.315 ***
	High school level (= 1; else = 0)	0.22	0.415	0.21	0.407	0.29	0.455	−0.085 ***
	Bachelor’s degree or above (= 1; else = 0)	0.12	0.33	0.07	0.261	0.43	0.495	−0.355 ***
Flow pattern	Intercountry within the city (= 1; other = 0)	0.2	0.398	0.20	0.396	0.21	0.407	−0.014 ***
	Intercity within the province (= 1; other = 0)	0.31	0.462	0.31	0.461	0.32	0.468	−0.02 ***
	Trans-provincial (= 1; other = 0)	0.49	0.5	0.50	0.500	0.47	0.499	0.034 ***
Attend medical insurance	Medical insurance (= 1; other = 0)	0.94	0.243	0.94	0.231	0.90	0.301	0.044 ***
Employment status	Employee (= 1; other = 0)	0.54	0.499	0.52	0.500	0.64	0.479	−0.124 ***
	Employer (= 1; other = 0)	0.08	0.272	0.08	0.268	0.09	0.292	−0.016 ***
	Independent Business (= 1; other = 0)	0.37	0.482	0.39	0.488	0.24	0.425	−0.153 ***
	Other (= 1; other = 0)	0.01	0.12	0.01	0.113	0.03	0.157	−0.013 ***
Long-term residence willingness (5 years)	Intend (= 1; other = 0)	0.6	0.491	0.58	0.493	0.67	0.471	−0.082 ***
	No plan (= 1; other = 0)	0.12	0.331	0.13	0.333	0.11	0.312	0.018 ***
	No idea (= 1; other = 0)	0.28	0.448	0.29	0.453	0.22	0.417	0.064 ***
Income	Low-income (= 1; other = 0)	0.04	0.194	0.04	0.198	0.03	0.166	0.013 ***
	Middle-income (=1; other = 0)	0.47	0.499	0.48	0.500	0.40	0.490	0.084 ***
	High-income (= 1; other = 0)	0.42	0.493	0.41	0.492	0.43	0.495	−0.019 ***
	Higher-income (= 1; other = 0)	0.07	0.263	0.06	0.244	0.14	0.348	−0.077 ***
Residence time (year)	Less than 3 years (= 1; other = 0)	0.43	0.496	0.44	0.496	0.42	0.493	0.023 ***
	3 to 6years (= 1; other = 0)	0.26	0.441	0.26	0.440	0.28	0.448	−0.016 ***
	More than 6 years (= 1; other = 0)	0.3	0.458	0.30	0.458	0.31	0.461	−0.007
Sample area	East (= 1; other = 0)	0.43	0.495	0.43	0.495	0.41	0.492	0.018 ***
	Central (= 1; other = 0)	0.18	0.386	0.19	0.391	0.15	0.356	0.039 ***
	West (= 1; other = 0)	0.32	0.468	0.32	0.468	0.33	0.472	−0.012 **
	Northeast (= 1; other = 0)	0.07	0.249	0.06	0.237	0.11	0.307	−0.045 ***
N		59,443		50,937		8506		

Notes: T-stats means the *t*-test of variables in different household registration, and when T exceeds a specific threshold, there is a significant difference between the two groups of samples. ***, **, and * indicate significance at the level of 1%, 5%, and 10%, respectively.

**Table 2:** Binary logistic results.

Dependent Variable		Y1				Y2			
Independent Variable		Factor	OR	OR 95% Confidence Interval		Factor	OR	OR 95% Confidence Interval	
				Minimum	Maximum			Minimum	Maximum
Hou		0.066 ** (0.027)	1.068	1.012	1.126	0.014 (0.063)	1.014	0.896	1.147
Gender		−0.081 *** (0.018)	0.922	0.891	0.955	−0.404 *** (0.041)	0.667	0.615	0.724
Age		0.021 *** (0.008)	1.022	1.006	1.038	0.064 *** (0.015)	1.066	1.035	1.099
Age* age		0.000 ** (0.000)	1	1	1	−0.001 *** (0.000)	0.999	0.998	0.999
Marriage		0.114 *** (0.029)	1.121	1.06	1.186	0.593 *** (0.058)	1.81	1.614	2.03
Education level	Middle school level	0.113 *** (0.028)	1.12	1.06	1.183	0.111 * (0.059)	1.117	0.995	1.254
	High school level	0.206 *** (0.033)	1.229	1.153	1.31	0.240 *** (0.071)	1.272	1.106	1.463
	Bachelor’s degree or above	0.16 *** (0.04)	1.174	1.086	1.269	0.239 *** (0.09)	1.27	1.065	1.514
Flow mode	Cross-city within the province	0.057 ** (0.024)	1.059	1.01	1.11	0.23 *** (0.058)	1.259	1.123	1.41
	Cross-province	−0.515 *** (0.023)	0.598	0.571	0.626	0.112 ** (0.056)	1.119	1.003	1.248
Employment status	Employer	0.124 *** (0.034)	1.132	1.06	1.209	−0.25 *** (0.075)	0.779	0.673	0.901
	Self-employed workers	0.115 *** (0.02)	1.121	1.079	1.165	−0.026 (0.046)	0.974	0.89	1.066
	Other	−0.025 (0.073)	0.976	0.845	1.126	0.259 (0.179)	1.296	0.913	1.839
Intended long-term residence in the local area (5 years)	No	−0.401 *** (0.029)	0.669	0.632	0.709	−0.126 ** (0.06)	0.881	0.783	0.992
	Not yet	−0.17 *** (0.021)	0.844	0.81	0.879	−0.204 *** (0.045)	0.816	0.747	0.891
Take part in health insurance		0.167 *** (0.037)	1.182	1.099	1.271	0.245 *** (0.071)	1.278	1.113	1.468
Length of residence	3-6 years	0.116 *** (0.022)	1.123	1.077	1.172	0.101 ** (0.05)	1.106	1.004	1.22
	More than 6 years	0.076 *** (0.022)	1.079	1.033	1.127	0.026 (0.05)	1.027	0.932	1.131
Income level	Middle	−0.009 (0.046)	0.991	0.905	1.085	0.288 *** (0.086)	1.334	1.127	1.578
	High	−0.099 ** (0.048)	0.905	0.825	0.994	0.427 *** (0.091)	1.533	1.283	1.833
	Higher	−0.123 ** (0.057)	0.884	0.791	0.988	0.664 *** (0.122)	1.943	1.53	2.467
Region	Central					0.316 *** (0.059)	1.372	1.222	1.541
	West					0.775 *** (0.053)	2.171	1.958	2.408
	Northeast					−0.008 (0.073)	0.992	0.86	1.144

Note: (1) Observations = 59443, reference group: Agricultural household registration, female, unmarried, primary school and below, city cross-county mobility, employees, intention to live in the local area long-term, 0 to 3 years of residence, low-income and eastern region (OR = 1.00); (2) the standard errors in parentheses indicate heteroscedastic robustness, * significant at the 10% level, ** significant at the 5% level, *** significant at the 1% level; and (3) to improve the fit of the model, the “region” variable is not added to the y1 regression.

**Table 3:** Binary logistic regression results for cross-entry.

Dependent Variable	Y1				Y2			
Independent Variable	Factor	Index of Regression Coefficients	OR 95% Confidence Interval		Factor	Index of Regression Coefficients	OR 95% Confidence Interval	
			Minimum	Maximum			Minimum	Maximum
Middle school*Hou	0.204 (0.148)	1.227	0.918	1.639	0.097 (0.302)	1.102	0.609	1.993
High school*Hou	0.098 (0.148)	1.103	0.826	1.474	0.004 (0.304)	1.004	0.554	1.821
Undergraduate/Specialist or above *Hou	−0.005 (0.149)	0.995	0.743	1.333	−0.001 (0.309)	0.999	0.545	1.829
Cross-city in the province *Hou	−0.13 * (0.067)	0.878	0.77	1.001	−0.037 (0.169)	0.964	0.692	1.341
Cross-provincial *Hou	0.056 (0.065)	1.057	0.931	1.201	−0.169 (0.154)	0.844	0.624	1.142
Middle income*Hou	0.232 (0.15)	1.261	0.94	1.691	−0.294 (0.311)	0.745	0.405	1.371
High income*Hou	0.328 ** (0.15)	1.388	1.034	1.863	−0.272 (0.315)	0.762	0.411	1.411
Higher income*Hou	−0.009 (0.164)	0.991	0.718	1.367	−0.444 (0.356)	0.641	0.319	1.289

Note: (1) Observations = 59443, reference group: Agricultural household registration, female, unmarried, primary school and below, city cross-county mobility, employee, intention to live in the local area long-term, 0 to 3 years of residence, low-income, and eastern region (OR = 1.00); (2)the standard errors in parentheses indicate heteroscedastic robustness, * significant at the 10% level, ** significant at the 5% level; (3) to improve the best fit of the model, the region variable was not added to the Y1 regression; and (4) other related variables are not listed due to space limitations.

**Table 4:** Average treatment effect on the treated (ATT) estimated by the Mars matching method.

Variable	Sample	Treated	Controls	Difference	S.E.	T-stat
y1	Unmatched	0.38349	0.35946	0.02403	0.0056	4.27
	ATT	0.38349	0.37036	0.01313	0.0076	1.73
y2	Unmatched	0.95532	0.95127	0.00405	0.0025	1.62
	ATT	0.95532	0.95518	0.00015	0.0032	0.05
